# IL-10 Regulates Viral Lung Immunopathology during Acute Respiratory Syncytial Virus Infection in Mice

**DOI:** 10.1371/journal.pone.0032371

**Published:** 2012-02-29

**Authors:** Jens Loebbermann, Corinna Schnoeller, Hannah Thornton, Lydia Durant, Nathan P. Sweeney, Martijn Schuijs, Anne O'Garra, Cecilia Johansson, Peter J. Openshaw

**Affiliations:** 1 Centre for Respiratory Infection/MRC and Asthma UK Centre, Respiratory Medicine, National Heart and Lung Institute, Faculty of Medicine, Imperial College, London, United Kingdom; 2 Division of Immunoregulation, MRC National Institute for Medical Research, The Ridgeway, Mill Hill, London, United Kingdom; National Jewish Health, United States of America

## Abstract

Interleukin (IL-) 10 is a pleiotropic cytokine with broad immunosuppressive functions, particularly at mucosal sites such as the intestine and lung. Here we demonstrate that infection of BALB/c mice with respiratory syncytial virus (RSV) induced IL-10 production by CD4^+^ and CD8^+^ T cells in the airways at later time points (e.g. day 8); a proportion of these cells also co-produced IFN-γ. Furthermore, RSV infection of IL-10^−/−^ mice resulted in more severe disease with enhanced weight loss, delayed recovery and greater cell infiltration of the respiratory tract without affecting viral load. In addition, IL-10^−/−^ mice had a pronounced airway neutrophilia and heightened levels of pro-inflammatory cytokines and chemokines in the bronchoalveolar lavage fluid. Notably, the proportion of lung T cells producing IFN-γ was enhanced, suggesting that IL-10 may act in an autocrine manner to dampen effector T cell responses. Similar findings were made in mice treated with anti-IL-10R antibody and infected with RSV. Therefore, IL-10 inhibits disease and inflammation in mice infected with RSV, especially during recovery from infection.

## Introduction

During acute lung infection, it is imperative that the host's inflammatory response is tightly regulated, enabling pathogen elimination but limiting the detrimental effects of inflammation on the gas exchange. An appropriate balance of anti-inflammatory and pro-inflammatory mediators (e.g. IL-10, TGF-β vs. TNF-α, IFN-γ, IL-6) is essential for a safe and effective antiviral immune response. Thus, an excessive IFN-γ response can lead to increased immunopathology, while exuberant IL-10 production can result in delayed pathogen clearance [1].

IL-10 can be produced by most cells of the immune system, including some regulatory T cells [2]. It has many immunosuppressive functions, inhibiting production and release of inflammatory cytokines by macrophages and monocytes and thus maintaining normal immune quiescence at mucosal sites [3], [4]. In addition, IL-10 is known to inhibit IL-12 production and thereby reduce Th1 development and IFN-γ production [5]. While inhibiting inflammatory signals, IL-10 also enhances phagocytic activity, which increases the removal of mediators and cell debris at sites of inflammation [6].

Epstein-Barr virus (EBV), cytomegalovirus (CMV) and several poxviruses encode IL-10 homologues [7], [8], probably in order to modulate host responses and possibly to recruit new target cells to the site of viral replication. Several parasites also induce IL-10 production, probably to allow persistence of infection [9]. Some bacteria (e.g. *Bordetella pertussis*) also promote IL-10 production [10].

Respiratory syncytial virus (RSV) is a major global cause of lower respiratory tract disease in children, especially those born prematurely, with low birth weight or suffering from cardiopulmonary disorders [11], [12]. RSV bronchiolitis in infancy is associated with recurrent wheeze in later life [13], and RSV also causes significant morbidity and mortality in immunosuppressed adults, those with heart or lung disease and in the elderly [14], [15]. Despite limited viral diversity, immunity to re-infection is only partial, allowing repeated infections with identical strains, which suggests that RSV may circumvent or evade normal immune defenses. Notably, IL-10 is not only detectable in the mouse model of RSV but also in nasopharyngeal secretions and serum of infants with severe bronchiolitis [16]–[18]. The most recent study has suggested a correlation between local IL-10 levels during the initial RSV infection and post-bronchiolitis wheeze, providing further evidence of the importance of IL-10 during human RSV bronchiolitis [18]. Furthermore, Hoebee *et al* associated risk of severe RSV bronchiolitis in infants with an IL-10 polymorphism, suggesting that IL-10 may be important in regulating RSV disease [19].

Two recent studies demonstrate a role for IL-10 in controlling immunopathology during influenza infection. While one shows that IL-10 prevents immunopathology and lethal disease [20], the other indicates that IL-10 has little impact on sublethal infection but inhibits beneficial Th17 responses during high-dose challenge [21]. Interestingly IL-10 also seems to play a crucial role in controlling disease severity in RSV infection [22], [23]. In both, acute influenza and RSV infection, CD4^+^ and CD8^+^ T cells were the major source of IL-10 and these cells were also able to coproduce IFN-γ [20], [21], [23]. Another recent study suggested CD4^+^ FoxP3^−^ and FoxP3^+^ cells to be the IL-10 producers during RSV infection [22].

To further investigate the role of IL-10 in pulmonary immune responses to RSV infection and provide further evidence to clarify the cellular source of IL-10, we examined the effects of experimental RSV infection in IL-10^−/−^ mice or mice treated with anti-IL-10 receptor (IL-10R) antibody. We found that IL-10 deficiency during RSV challenge did not affect viral load, but led to markedly increased disease severity with enhanced weight loss, delayed recovery and a greater influx of inflammatory cells into the lung and airways and enhanced release of inflammatory mediators. Interestingly, we identified effector CD4^+^ and CD8^+^ T cells as the main cellular source of IL-10, and showed that most of those cells co-produced IFN-γ. Our results therefore confirm IL-10 to be a key anti-inflammatory cytokine responsible for immune regulation in the lung during acute RSV infection of mice, with Foxp3 negative CD4^+^ and CD8^+^ T cells being the main contributors. These data emphasize the role that defective immunoregulation may play in the pathogenesis of severe viral lung disease.

## Results

### IFN-γ and IL-10 co-production by CD4^+^ and CD8^+^ T cells during RSV infection

**Figure 1:**
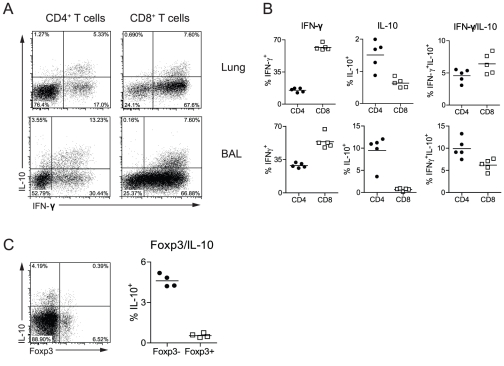
RSV infection induces IL-10/IFN-γ double-producing CD4^+^ and CD8^+^ T cells in the lung and airways. BALB/c mice were infected with 10^6^ PFU RSV i.n. (day 0). CD3^+^ gated, CD4^+^ T cells and CD8^+^ T cells from lungs and airways on day 8 post RSV infection were analyzed by flow cytometry. (A) Representative plots of intracellular IFN-γ and IL-10 expression by CD4^+^ and CD8^+^ T cells from lung and BAL after a 3 hr PMA/ionomycin restimulation are shown. (B) Quantifications of the frequencies of single producing IFN-γ or IL-10 T cells, and IL-10/IFN-γ double producing CD4^+^ and CD8^+^ T cells on day 8 post RSV infection are shown. (C) CD3^+^CD4^+^ gated T cells and from lungs on day 8 post RSV infection were analyzed for expression of IL-10 and Foxp3 by flow cytometry. A representative plot and quantifications are shown. The data is representative of three independent experiments each with 4–5 mice per group.

To demonstrate the presence and origin of IL-10 during RSV infection, BALB/c mice were infected with human RSV A2. Cells from the lungs and airways were analyzed on day 4 and 8 post RSV infection for IFN-γ and IL-10 expression using flow cytometry. T cells expressed negligible amounts of IL-10 or IFN-γ in the lung and bronchoalveolar lavage fluid (BAL) on day 4 post RSV infection (not depicted), but both CD4^+^ and CD8^+^ T cells from the lung or airways frequently expressed IFN-γ and IL-10 on day 8 post infection (Fig. 1A and B). Interestingly, only CD8^+^ T cells that expressed high levels of IFN-γ co-expressed IL-10 resulting in 6% of CD8 IFN-γ producing cells in lung and BAL co-producing IL-10 and less than 1% IL-10 single producers. In contrast, up to 8% of CD4 cells in the BAL were IL-10 single-producers, in addition to 10% of CD4^+^ T cells that were IL-10/IFN-γ double-producers (Fig. 1 A and B). Despite the recent demonstration that Foxp3^+^ Tregs are the main producers of IL-10 in RSV disease [22], the majority of our IL-10 expressing CD4^+^ T cells were Foxp3^−^ (Fig. 1 C), confirming data highlighting CD4 and CD8 effector cells rather than Foxp3^+^ cells as a crucial source for immunoregulatory IL-10 [23].

### Lack of IL-10 increases cellular influx into the lungs and delays recovery during RSV infection

**Figure 2:**
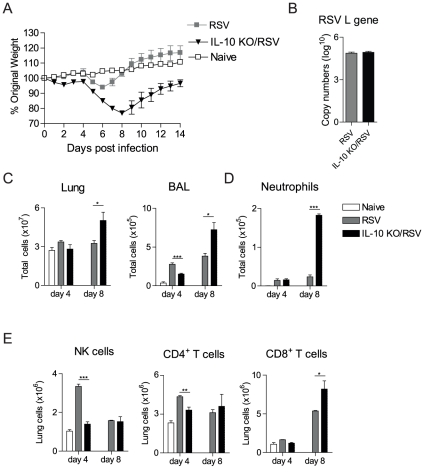
Lack of IL-10 increases cellular influx into the lungs and delays recovery during RSV infection. BALB/c mice and IL-10^−/−^ mice were infected with 10^6^ PFU RSV i.n. (day 0). (A) Illness was monitored daily by changes in weight for 14 days after RSV infection; percentage of original weight (day 0) is shown. (B) Copies of the RSV L gene were quantified in the lung on day 4 post infection using qPCR. (C) Total numbers of cells in the lung and BAL were enumerated on day 4 and 8 from naïve or RSV infected mice. (D) Total numbers of neutrophils in the BAL were quantified using differential cell counting of H&E stained cytospins slides on day 4 and 8 post infection. (E) Total numbers of NK cells (CD3^−^ NKp46^+^) and CD3 gated CD4^+^Foxp3^−^ and CD8^+^ T cells in the lung were quantified using flow cytometry on day 4 and 8 post RSV infection. Error bars indicate the SEM. The data is representative of three independent experiments with n = 4–5 mice per group.

Since RSV infection induced IL-10 expression by T cells, we wished to test the effects of IL-10 deficiency on the course of disease. Therefore, BALB/c WT or IL-10^−/−^ mice were infected with RSV; mice were monitored for disease and daily weight loss and lung/BAL cells analyzed on day 4 and 8 post infection. BALB/c IL-10^−/−^ mice infected with RSV showed increased and sustained weight loss and delayed recovery compared to control BALB/c mice (Fig. 2 A). The absence of IL-10 did not affect viral load in the lung on day 4 post infection (Fig. 2 B), the time of peak of viral load [24]. However, IL-10-deficient mice had increased total cell numbers in the lung and BAL on day 8 post RSV infection (Fig. 2 C) while total cell numbers in the BAL on day 4 post infection were decreased in IL-10^−/−^ mice compared to controls (Fig. 2 C). Notably, the absence of IL-10 led to an increased efflux of neutrophils in the BAL on day 8 post RSV infection, but made no difference on day 4 (Fig. 2 D). No eosinophils were detected at any time point in any of the groups (data not depicted). Unexpectedly, both NK cells and CD4^+^Foxp3^−^ T cells were scarcer in the lung on day 4 post RSV infection in the IL-10^−/−^ mice (Fig. 2 E). Furthermore, while the number of CD4^+^Foxp3^−^ T cells was similar between the groups on day 8 post infection (data not depicted), CD8^+^ effector T cells were elevated in the lungs of IL-10^−/−^ mice (Fig. 2 E).

Thus, the lack of IL-10 during RSV infection manifests itself as a markedly increased recruitment of inflammatory CD8^+^ T cells to the lung, enhanced airway neutrophilia and increased disease severity in the later stage of RSV disease.

### Lack of IL-10 increases IFN-γ expression in T cells after RSV infection

**Figure 3:**
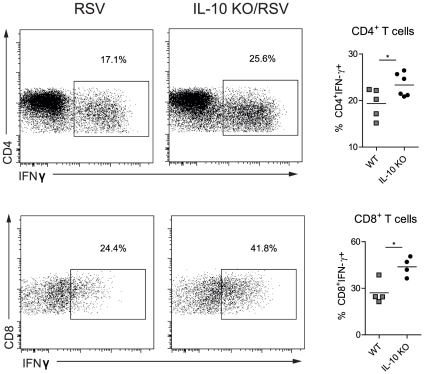
Increased frequency of IFN-γ-expressing T cells in IL-10^−/−^ mice after RSV infection. BALB/c mice were infected with 10^6^ PFU RSV i.n. (day 0). CD3^+^ gated, CD4^+^ and CD8^+^ T cells from lungs on day 8 post RSV infection were analyzed by flow cytometry. Representative plots of intracellular IFN-γ expression after a 3 hr PMA/ionomycin restimulation are shown. In addition, quantifications of the frequencies of IFN-γ expression in CD4^+^ T cells and CD8^+^ T cells are shown. The data are representative of three independent experiments with n = 4–5 mice per group.

IL-10 production by Th1 cells has been reported to act in an autocrine fashion to dampen IFN-γ responses in other pathological settings [25]. Hence, it was of interest to investigate whether IL-10 similarly regulated IFN-γ expression during RSV infection. We therefore harvested lung cells from RSV- infected WT or IL-10-deficient mice and measured IFN-γ expression in T cells following a 3 h restimulation with PMA and ionomycin. Notably, the lack of IL-10 significantly increased the frequency of IFN-γ^+^ CD8^+^ and CD4^+^ T cells in the lung on day 8 post infection (Fig. 3), supporting the conclusion that IL-10 is important for dampening the activation of inflammatory T cells during RSV infection.

### Blocking IL-10R signalling increases cellular influx into the lungs and delays recovery during RSV infection

Using IL-10^−/−^ mice, we have shown that mice suffer more severe disease during RSV infection. IL-10^−/−^ mice can develop a spontaneous colitis by 12 wks of age [26] and though we used young IL-10^−/−^ mice (<10 wks), we wanted to confirm our findings and rule out secondary adverse effects due to congenital IL-10-deficiency. Therefore, short-term IL-10 depletion was achieved by injecting WT BALB/c mice with anti-IL-10 receptor (αIL-10R) antibody. Mice were either treated with anti-IL-10R, rat IgG control antibody or left untreated before infection with RSV. As an index of disease severity, individual body weights were recorded daily.

**Figure 4:**
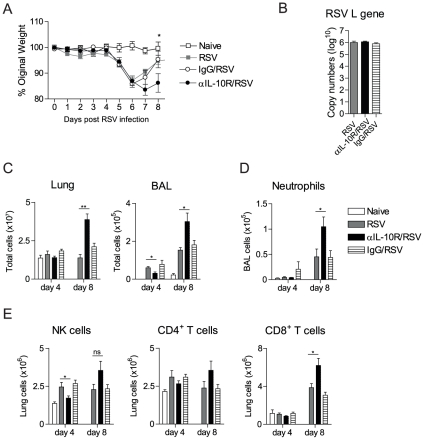
Blocking IL-10R signalling increases cellular influx into the lungs and delays recovery during RSV infection. BALB/c mice were infected with 10^6^ PFU RSV i.n. (day 0). Where indicated, mice were injected with anti-IL-10R antibody on day -1 (i.p.), 3 (i.p. and i.n) and 6 (i.p.) post RSV infection. Control groups were injected with rat IgG. (A) Illness was monitored daily by changes in weight for 8 days after RSV infection; the percentage of original weight is shown. (B) Viral titer was measured in the lung on day 4 post infection by quantifying RSV L gene copies by qPCR. (C) Total numbers of cells in the lung and BAL were enumerated on day 4 and 8 from naïve or RSV infected mice. (D) Total numbers of neutrophils in the BAL were quantified using differential cell counting of H&E stained cytospins slides on day 4 and 8 post infection. (E) Total numbers of NK cells (CD3^−^ NKp46^+^) and CD3-gated, CD4^+^Foxp3^−^ and CD8^+^ T cells in the lung were quantified using flow cytometry on day 4 and 8 post RSV infection. Error bars indicate the SEM. The data are representative of two independent experiments with n = 4–5 mice per group.

In accordance with the IL-10-deficient mice, anti-IL-10R treated mice infected with RSV showed increased and sustained weight loss and delayed recovery compared to IgG-treated or untreated control BALB/c mice (Fig. 4 A). While IL-10R blockade did not change viral load in the lungs on day 4 post infection (Fig. 4 B), it did lead to an increase in the total cell numbers in the lung and BAL on day 8 (Fig. 4 C), as seen in IL-10^−/−^ mice. Interestingly, mice treated with anti-IL-10R antibody also displayed a decrease in total cell numbers in the BAL on day 4 post infection compared to control BALB/c mice (Fig. 4 C). Notably, anti-IL-10R treatment also led to an increase in total numbers of neutrophils in the BAL on day 8 post RSV infection (Fig. 4 D), while no differences were detected on day 4 (Fig. 4 D). As seen previously in the IL-10^−/−^ mice, NK cells in mice treated with anti-IL-10R antibody were reduced in the lung on day 4 post RSV infection (Fig. 4 E). Furthermore, while CD4^+^Foxp3^−^ T cells numbers were similar between the two groups of mice on day 8 post infection (Fig. 4 E), CD8^+^ effector T cell populations were elevated in the lungs of mice treated with anti-IL-10R compared to control or IgG isotype-treated mice (Fig. 4 E).

Therefore, short-term blockade of IL-10R signalling during RSV infection leads to markedly increased recruitment of inflammatory cells to the lung and greater disease severity comparable to IL-10^−/−^ mice, suggesting no crucial secondary adverse effects due to congenital IL-10 deficiency in this setting.

### Lack of IL-10 increases chemokine and cytokine production in the airways during RSV infection

**Figure 5:**
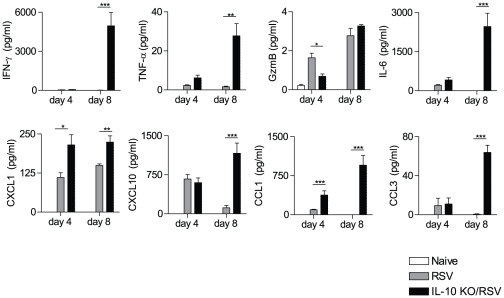
Lack of IL-10 increases chemokine and cytokine production in the airways during RSV infection. BAL samples from BALB/c and IL-10^−/−^ mice were analyzed for indicated cytokines and chemokines on day 4 and 8 after RSV infection using Luminex. A group of naive BALB/c controls are included in the day 4 data. Error bars indicate the SEM. The data are pooled from two independent experiments with n = 4–5 mice per group.

Chemokine and cytokine expression plays an important role in the recruitment and activation of different cell types during RSV infection [27]. To elucidate the role of IL-10 in this response we measured the levels of mediators in the BAL fluid from naïve mice, WT or IL-10^−/−^ mice at day 4 and 8 post RSV infection (Fig. 5). The levels of CXCL1, CXCL10, CCL1, CCL3, IFN-γ, TNF-α and IL-6 were significantly increased in the lungs of RSV-infected IL-10-deficient mice compared to control mice on day 8 post infection (Fig. 5). Interestingly, the level of granzyme B (GzmB) was decreased in BAL fluid of IL-10^−/−^ mice on day 4 post RSV infection, possibly reflecting the reduction in NK cells (a major source of GzmB [28]) in the airways at this time point.

Overall, these findings suggest that IL-10 acts to limit the production of diverse inflammatory cytokines and chemokines during RSV infection, thereby restricting pathology in RSV disease.

## Discussion

We used two different *in vivo* approaches to examine the contribution of IL-10 in controlling virally induced lung inflammation: IL-10^−/−^ mice and short-term treatment of WT mice with anti-IL-10R antibody. We found that lung or airway CD4^+^ and CD8^+^ effector T cells were the main source of IL-10, and the majority of these cells were able to co-produce IFN-γ. IL-10R blockade or IL-10 gene deletion caused increased and sustained weight loss after RSV infection, accompanied by an increase in inflammation and cell recruitment to the lung and airways. Importantly, the general increase in cell recruitment was reflected in recruitment of neutrophils and CD8^+^ T cells.. Together, these findings highlight a central role for IL-10 in controlling harmful immunopathology.

We confirmed that both CD4^+^ and CD8^+^ effector T cells were able to coproduce IL-10 and IFN-γ during RSV infection, in line with recent studies [20], [21], [23]. Interestingly, we also demonstrated CD4^+^ T cells (but not CD8^+^ T cells) that only expressed IL-10, a phenotype previously attributed to the regulatory subset termed Tr1 cells [29]. While the significance of IL-10^+^ IFN- γ^−^ CD4^+^ T cells in RSV infection is uncertain, Cardone *et al* recently demonstrated in humans that IL −10 expression can occur through a negative feedback loop via CD46 engagement, which subsequently induces “intrinsic” IL-10 expression in cells that previously expressed IFN-γ and reveal a Tr1 phenotype [30]. Sun *et al* have also recently demonstrated a Th1 phenotype for all IL-10 expressing CD4^+^ T cells during RSV infection, but did not distinguish CD4^+^ IL-10^+^ T cells from CD4^+^ IL-10^+^ IFN-γ^+^ co-producers during RSV infection [23]. Thus, the phenotype and function of CD4^+^ IL-10^+^ IFN-γ^−^ T cells in the lungs and airways during RSV infection remain to be elucidated.

Several studies have indicated that Foxp3^+^ Tregs are a potential source of IL-10 [31]–[34]. However, in contrast to the findings of Weiss *et a*, [22] , we did not find that Foxp3^+^ Tregs were a major source of IL-10 . However, we focussed on IL-10 expression by T cells and therefore we cannot be certain that IL-10 is not produced by other cell types (e.g. epithelial cells, dendritic cells, macrophages or B cells), especially at early time points after RSV infection.

Interestingly, the absence of IL-10 also altered the NK cell response in the lung after RSV infection. We found a decrease in NK cells numbers and reduced GzmB expression by NK cells in the lungs and airways on day 4 post RSV infection. Stacey *et al* recently demonstrated that IL-10R blockade during acute murine CMV infection resulted in impaired NK cell responsiveness and proposed that IL-10 acts to promote NK cell activation and survival in the lung [35]. However, in our model viral load remained unchanged, implicating that any defect in NK survival or activation did not affect their ability to kill virus-infected cells.

From our findings that IL-10 is essential for controlling the inflammatory response during acute viral lung infection, we propose that IL-10 released by T cells acts in an autocrine fashion on T cells directly to regulate their activation status. Our results demonstrate that in the absence of IL-10, T cells have increased levels of IFN-γ production, suggesting that IL-10 is necessary to “self-regulate” the T cell response in the lungs and airways at the peak of the adaptive immune response. These findings were confirmed in a recent study by Sun *et al* using CD4-cre IL-10Rα fl/fl mice. In this study, the specific disruption of IL-10 signalling in T cells led to increased weight loss and IFN-γ levels in the airways during RSV infection, suggesting a novel autocrine function of IL-10 in the onset of acute viral lung infection [23]. Pils *et al* proposed a similar autocrine feedback loop in macrophages and neutrophils in the model of LPS endotoxemia [36]. The molecular mechanism and triggers underlying autocrine IL-10 production by monocytes and effector T cells remains to be elucidated.

Conversely, IFN-γ has also been shown to suppress inflammation in RSV challenged previously vaccinated mice [37]. Although IFN-γ can be beneficial in limited amounts in RSV-infected mice its production and anti-inflammatory effects need to be tightly regulated by expression of IL-10, possibly through an autocrine loop. Therefore, T cells in IL-10 deficient mice are not able to self-regulate their IFN-γ production and this could contribute to the elevated immunopathology detected in IL-10 deficient mice in the later stages of RSV infection.

Our demonstration that IL-10 plays a pivotal role in controlling immunopathology driven lung inflammation *in vivo* and the further highlighting of Foxp3^−^ effector T cells as main lymphocytes producing it is an important step in elucidating the mechanisms by which the fine balance between clearing virus and controlling immune responses is achieved in the respiratory tract, allowing normal gas exchange to be maintained in the face of viral attack. From these findings, we speculate that selectively enhancing IL-10 production, release or signalling and special consideration of IL-10/IFN-γ double producing CD4 and CD8 effector cells could potentially be used as a therapeutic strategy to modulate inflammation in acute viral lung infections, with little risk of enhancing viral load. Combinations of antiviral medication with immune modulation are especially promising as a future therapeutic approach.

## Materials and Methods

### Ethics statement

All mouse experiments were ethically approved by the Imperial College Central Biological Services (CBS) ethics committee performed in accordance with approved UK Home Office guidelines (Project Licence No. PPL 70/6785).

### Mice, virus stocks and infection

Six to ten week old BALB/c IL-10^−/−^ mice and BALB/c WT mice (Harlan, UK) were maintained in pathogen-free conditions and all experiments were performed in accordance with approved UK Home Office guidelines.

Plaque-purified human RSV (type A2 strain from the ATCC) was grown in HEp-2 cells [38]. Age- and sex-matched mice were lightly anesthetized and infected intranasally (i.n.) with 10^6^ PFU RSV in 100 µl.

### Anti-IL-10R antibody treatment

In indicated groups *in vivo* treatment with anti-IL-10R antibody (1B1.3a) was performed. Mice were injected intraperitoneally on day -1, 3 and 6 with 1 mg antibody and intranasally on day 3 with 0.15 mg Ab, and were infected intranasally with 10^6^ PFU RSV in 100 µl on day 0. Control groups were treated with equal amounts of Rat IgG antibody. Both anti-IL10R antibody and anti-IgG antibody were kindly provided by Anne O'Garra.

### Cell collection and preparation

Bronchoalveolar lavage (BAL) was carried out using 1 ml PBS containing 12 mM Lidocaine and flushing through the trachea 3 times. In order to obtain single cell suspensions, LNs were mashed through a cell strainer and lungs were incubated with Collagenase D (50 µg/ml, Sigma) and processed using the gentleMACS dissociator (Miltenyi Biotech) according to the manufacturer's protocol. Total cell counts were determined by flow cytometry using CountBright absolute counting beads (Invitrogen) and dead cells were excluded using 7-amino-actinomycin D (7-AAD, Sigma). For determination of cellular composition in the BAL, cells were transferred onto a microscope slide (Thermo Scientific, UK) using a cytospin centrifuge and stained with hematoxylin and eosin (H&E; Reagena, Gamidor, UK).

### Flow cytometry

For flow cytometry analysis the LIVE/DEAD Fixable Red Dead cell stain kit (Invitrogen) was used to exclude dead cells. Cells were incubated in FACS staining buffer (PBS containing 1% BSA and 5 mM EDTA) with FcγIII/II receptor antibody (BD) and the following antibodies (from BD Biosciences unless otherwise stated): V450 or PE conjugated anti-mNKp46/NCR1, PE-Cy7 or V500 conjugated anti-CD3 (145-2C11), Pacific Blue or APC-H7 conjugated anti-CD4 (RM4-5), Alexa Fluor 700 conjugated anti-CD8α (53-6.7), CD3 APC-Cy7 (17A2) (eBioscience), CD4 Alexa 700 (RM4-5) (eBioscience), CD8 eFluor 450 (53-6.7) (eBioscience), CD49b PE-Cy7 (DX5) (eBioscience), CD4 PerCp Cy5.5 (RM4-5) and CD25 PE (3C7). Intracellular staining for Foxp3 and GzmB was performed using the Foxp3 staining kit (eBioscience) following manufacturer's recommendations with the following antibodies: APC or FITC conjugated anti-Foxp3 (FJK-16s, eBioscience) and APC conjugated anti-human GzmB (GB12, Caltag, Invitrogen). Anti-human GzmB antibody cross-reacts with mouse GzmB [39]. For intracellular IFN-γ and IL-10 staining, cells were stimulated with 100 ng/ml PMA and 1 µg/ml Ionomycin in complete RPMI (RPMI 1640 medium supplemented with 10% fetal bovine serum, 2 mM L-glutamine, 100 U/ml penicillin and 100 µg/ml streptomycin). After 1 h incubation, monensin (Golgi Stop, BD) was added. Following 2 additional hours of incubation, cell surface staining was followed by intracellular cytokine staining using the Fix/Perm Kit (BD) with Percp Cy 5.5 anti-IFN-γ, PE IL-17 (TC11-18H10) and APC or FITC conjugated IL-10 (JES5-16E3, eBioscience) antibodies. Cells were acquired on a LSR II (BD, United Kingdom) with data analyzed using Flow Jo software (v7.6.5). Cells were gated for live cells, singlets and lymphocytes before analysis for indicated markers.

### Chemokine and cytokine detection

Chemokines and cytokines were quantified by a 24-plex Luminex kit performed according to the manufacturer's instructions (Biolegend) and data acquired with a Luminex 100 (Applied Cytometry systems). The concentration of cytokines in each sample was determined according to the standard curve using the software Starstation. GzmB levels in the BAL were measured by ELISA following manufacturer's recommendations (R&D).

### Real time quantitative PCR

Total RNA was extracted from homogenized lung tissue using the Stat60-RNA extraction reagent (AMS Biotechnology Ltd., United Kingdom) and transcribed to cDNA by using random hexamers and Omniscript reverse transcriptase (Qiagen, United Kingdom). TaqMan real time quantitative PCR was performed with primers and probes for RSV L gene and 18S rRNA housekeeping gene as control (Invitrogen if not otherwise stated) (RSV L gene forward primer 900 nM, (5′-GAACTCAGTGTAGGTAGAATGTTTGCA-3′), RSV L gene reverse primer 300 nM (5′-TTCAGCTATCATTTTCTCTGCCAAT-3′), L gene probe 175 nM (5′-TTTGAACCTGTCTGAACATTCCCGGTT -3′) FAM TAMRA (Eurofins MWG Operon), 18S forward (5′-CGCCGCTAGAGGTGAAATTCT-3′), 18S reverse (5′-CATTCTTGGCAAATGCTTTCG-3′), 18S probe (5′-ACCGGCGCAAGACGGACCAGA-3′) FAM TAMRA, Erofins MWG Operon) as previously described [38].

### Statistical analysis

Results are presented as mean ± SEM. Statistical significance was determined using a two-tailed, unpaired Student's t test (*p<0.05, **p<0.01, ***p<0.001). Values of p<0.05 were considered significant (Prism software; Graph-Pad Software Inc.).
